# Emergent 3D Fermiology and Magnetism in an Intercalated Van der Waals System

**DOI:** 10.1002/advs.76533

**Published:** 2026-07-27

**Authors:** Luigi Camerano, Emanuel A. Martínez, Victor Porée, Laura Martella, Dario Mastrippolito, Debora Pierucci, Franco D'Orazio, Polina M. Sheverdyaeva, Paolo Moras, Enrico Della Valle, Tianlun Yu, Moritz Hoesch, Craig M. Polley, Thiagarajan Balasubramanian, Alessandro Nicolaou, Luca Ottaviano, Vladimir N. Strocov, Gianni Profeta, Federico Bisti

**Affiliations:** ^1^ Department of Physical and Chemical Sciences University of L'Aquila L'Aquila Italy; ^2^ CNR‐SPIN L'Aquila L'Aquila Italy; ^3^ CNRS Institut des Sciences Chimiques de Rennes‐UMR6226 Univ Rennes Rennes France; ^4^ Synchrotron SOLEIL L'Orme des Merisiers Gif‐sur‐Yvette France; ^5^ CNRS, Institut des NanoSciences de Paris Sorbonne Université Paris France; ^6^ CNR‐Istituto di Struttura della Materia (CNR‐ISM) Trieste Italy; ^7^ Swiss Light Source Paul Scherrer Institute Switzerland; ^8^ Deutsches Elektronen‐Synchrotron DESY Hamburg Germany; ^9^ MAX IV Laboratory Lund University Lund Sweden

**Keywords:** condensed matter physics, electronic structure, fermi level, inelastic scattering, lattice constant, magnetism, van der Waals force

## Abstract

Intercalation of magnetic atoms into van der Waals materials provides a versatile platform for tailoring unconventional magnetic properties. However, its impact on electronic dimensionality and exchange mechanisms remains poorly understood. Using Fe‐intercalated ${\mathrm{TaS}}_{2}$ as a model system, we combine x‐ray absorption and resonant inelastic scattering with angle‐resolved photoemission and first‐principles calculations to reveal that intercalation reshapes the host electronic structure. We identify a spin‐polarized intercalant‐host hybridized band with pronounced out‐of‐plane dispersion crossing the Fermi level, providing an itinerant channel for interlayer magnetic exchange. This mechanism explains the breakdown of a purely atomic picture and establishes a direct link between lattice geometry, electronic dispersion, and magnetic order. Our findings demonstrate that intercalant‐induced itinerancy enables tunable interlayer coupling in otherwise layered magnets, offering a general microscopic framework for engineering magnetic dimensionality in a broad class of intercalated vdW materials.

## Graphical abstract

An emergent three‐dimensional electronic state is uncovered in an intercalated van der Waals system. A spin‐polarized intercalant‐host hybridized band crossing the Fermi level provides an itinerant channel for interlayer exchange, highlighting how electronic dispersion governs magnetic dimensionality.

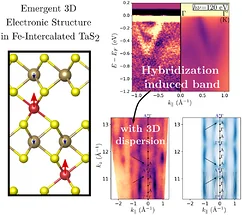

## 1 Introduction

The interaction between localized magnetic moments and conduction electrons gives rise to unconventional physical states showing exotic magnetism [1, 2], topological superconductivity [3, 4] and Kondo physics [5, 6]. The intercalation of magnetic atoms in layered metallic van der Waals (vdW) transition metal dichalcogenides (TMDs) (Figure 1a) provides a multifunctional platform to engineer a variety of quantum states arising from this interaction. Some examples are: highly tunable magnetic states in ${\mathrm{Fe}}_{1/3+x}{\mathrm{NbS}}_{2}$ [7, 8, 9], non‐coplanar magnetic order giving rise to spontaneous Hall effect in ${\mathrm{Co}}_{1/3}{\mathrm{TaS}}_{2}$ and ${\mathrm{Co}}_{1/3}{\mathrm{NbS}}_{2}$ [10, 11, 12], switchable helical spin texture in ${\mathrm{Ni}}_{1/3}{\mathrm{NbS}}_{2}$ [13], chiral helimagnetic states in ${\mathrm{Cr}}_{1/3}{\mathrm{TaS}}_{2}$ and ${\mathrm{Cr}}_{1/3}{\mathrm{NbS}}_{2}[$ 14, 15, 16], tunable Ising ferromagnetism and topological Hall effect in ${\mathrm{Fe}}_{x}{\mathrm{TaS}}_{2}$ [17, 18], controllable nematic states in ${\mathrm{Co}}_{1/3}{\mathrm{TaS}}_{2}$ [19, 20] and g‐wave altermagnetism in ${\mathrm{Co}}_{1/4}{\mathrm{NbSe}}_{2}$ [21, 22, 23, 24]. All these states are ultimately stabilized through the delicate balance between Kondo and Ruderman–Kittel–Kasuya–Yosida (RKKY) interactions, modulated by the concentration of the transition metal (TM) intercalant, which in turn tunes the doping of the TMD metallic state. A particularly interesting platform to study the competition between these interactions is Fe‐intercalated ${\mathrm{TaS}}_{2}$ that exists at different Fe concentrations (x) [25, 26, 27, 28, 29, 30]. While at very low concentration (x<0.05) Fe intercalation rises the superconductive critical temperature of the host ${\mathrm{TaS}}_{2}$ [26, 27], for 0.25<x<0.40 it induces ferromagnetism with tunable critical temperatures. Beyond x>0.4 an antiferromagnetic phase emerges [25, 28]. Interestingly, at concentrations x=1/4 and x=1/3, air‐stable ordered reconstructions are observed in these compounds [29, 30, 31, 32]. In particular, at x=1/4 a 2×2 reconstruction is observed with a ferromagnetic Curie temperature of $T_{c}$ = 135 K, whereas at x=1/3 the system presents a $\sqrt{3}\times \sqrt{3}$ rotated by 30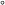 reconstruction ($\sqrt{3}\times \sqrt{3}R30^{\circ }$) with a $T_{c}$ = 70 K [25] and large anisotropic magnetoresistance [33, 34]. Additionally, magnetic properties were found to exhibit a pronounced dimensional dependence [35], and signatures of non‐ideal 2D behavior have emerged [36]. Understanding this rich phase diagram requires a detailed comprehension of how intercalant atoms influence the electronic structure of the TMD, as well as of the role played by the hybridization between the magnetic atoms and the host lattice.

**FIGURE 1:**
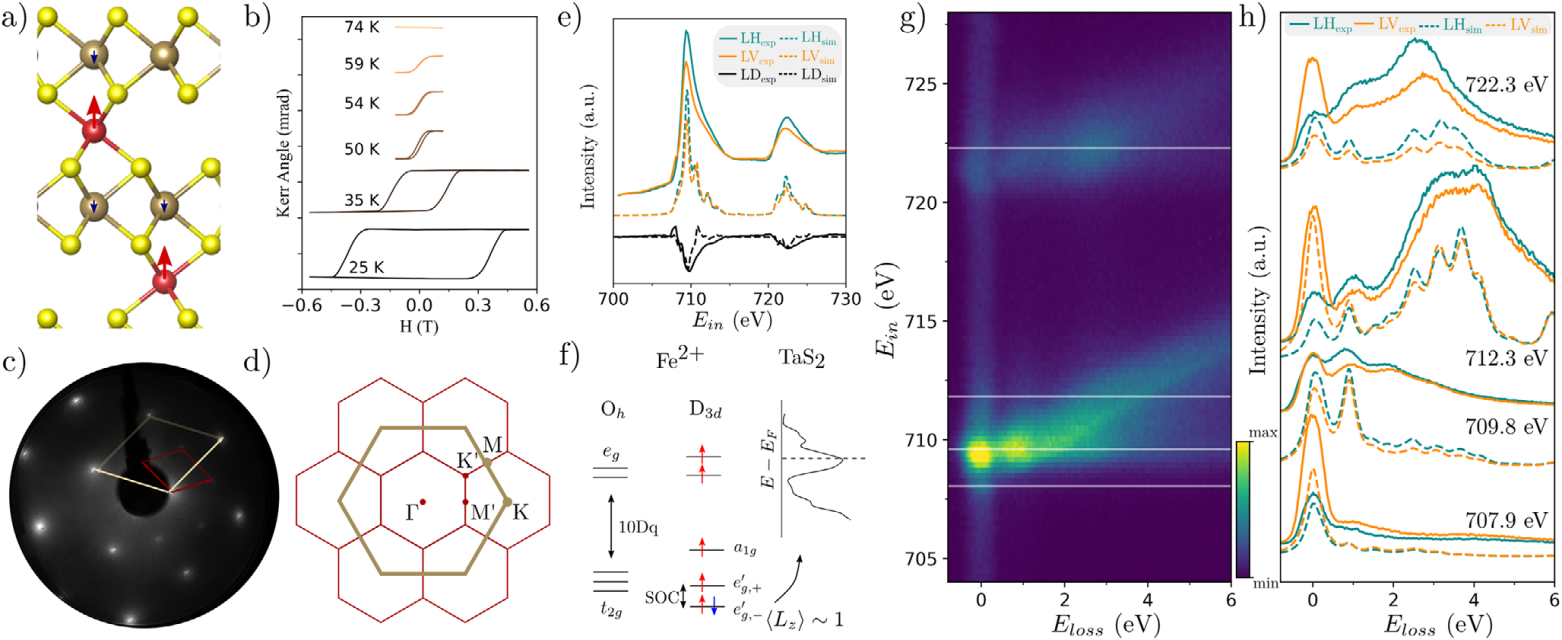
(a) Unit cell of ferromagnetic ${\mathrm{Fe}}_{1/3}{\mathrm{TaS}}_{2}$ with c‐axis aligned along the z‐direction. The spins on the Fe ions are reported in red, whereas those on the Ta ions are in blue. (b) Field dependent MOKE curves at various temperatures of ${\mathrm{Fe}}_{1/3}{\mathrm{TaS}}_{2}$ using a wavelength of 625 nm. The vertical tick marks are spaced by 10 mrad. (c) LEED spots acquired with 200 eV highlighting the $\sqrt{3}\times \sqrt{3}R30^{\circ }$ reconstruction. (d) Sketch of the reciprocal space in which we reported the $\sqrt{3}\times \sqrt{3}R30^{\circ }$ ordered reconstruction induced by the Fe intercalation. (e) Fe $L_{2,3}$ absorption spectra for linearly polarized incident radiation along with the corresponding linear dichroism. The experimental and simulated spectra are displayed in continuous and dashed lines, respectively. (f) Energy level scheme showing $O_{h}$ symmetry breaking into $D_{3d}$ symmetry and the filling of Fe‐d orbitals according to the model used to simulate the XAS/RIXS and ${\mathrm{TaS}}_{2}$ density of states near the Fermi level simulated with HSE06 functional highlighting ${\mathrm{Fe}}^{2+}$ embedded in a metallic matrix. (g) RIXS map of the Fe $L_{2,3}$ edges collected using circularly polarized incident radiation. (h) Experimental (continuous lines) and simulated (dashed lines) RIXS spectra acquired using linearly polarized incident light. The corresponding incident energies are given above each curve and are indicated by the white horizontal lines in (g).

To address these issues, we use ${\mathrm{Fe}}_{1/3}{\mathrm{TaS}}_{2}$ as a model system and combine X‐ray absorption spectroscopy (XAS) and resonant inelastic X‐ray scattering (RIXS) to probe the local electronic environment, together with angle‐resolved photoemission spectroscopy (ARPES) supported by first‐principles modeling and multiplet calculations. While previous studies established the Ising‐like character of the Fe magnetic moments and discussed magnetic coupling within an in‐plane RKKY framework [18] with Fe intercalant acting as an electron donor, we demonstrate the emergence of an intrinsically three‐dimensional, spin‐polarized itinerant band induced by intercalation that qualitatively reshapes the electronic structure of the host matrix giving rise to a pronounced $k_{z}$ dispersion. We discover that this band crosses the Fermi level and provides an efficient channel for mediating interlayer magnetic exchange. Consequently, the carrier‐mediated exchange is expected to acquire a significant out‐of‐plane component, corresponding to a three‐dimensional RKKY interaction rather than a purely two‐dimensional one. Because this mechanism relies only on the presence of metallic van der Waals hosts and symmetry‐allowed hybridization with intercalant orbitals oriented along the out‐of‐plane direction, it is expected to be generic across a broad class of intercalated dichalcogenides. Our results show that intercalant‐induced itinerancy acts as a tunable pathway for modifying lattice geometry, electronic dispersion, and magnetic order in van der Waals magnets, offering a unified microscopic perspective for engineering interlayer magnetism.

## 2 Atomic Structure and Magnetic Order

Bulk 2H‐${\mathrm{TaS}}_{2}$ is formed by stacked 1H‐${\mathrm{TaS}}_{2}$ monolayers (MLs) in an ABA sequence related by a glide‐mirror symmetry (Figure 1a). The 1H‐${\mathrm{TaS}}_{2}$ ML consists of a hexagonal lattice of Ta atoms in trigonal prismatic coordination with the chalcogen [37]. Fe intercalation in the interlayer spacing of 2H‐${\mathrm{TaS}}_{2}$ induces ferromagnetism (see Figure 1a,b). This is demonstrated by our temperature dependent magneto‐optical Kerr effect (MOKE) hysteresis loop at various temperature that show a ferromagnetic transition between 59 and 74 K, consistent with previous experimental reports Figure 1b [25, 29, 30, 32]. Moreover, the Low Energy Electron Diffraction (LEED) pattern (taken at $E_{kin}$ = 200 eV, Figure 1c) shows presence of 1 × 1 spots and an additional periodicity compatible with $\sqrt{3}\times \sqrt{3}R30^{\circ }$ reconstruction, indicated as a red hexagon, as shown in Figure 1d where we report a sketch of the reciprocal Brillouin Zone (BZ) of both the unit cells. We indicate with prime letters the BZ of the superstructure. This superstructure is typical of the x=1/3 concentration of Fe in pristine 2H‐${\mathrm{TaS}}_{2}$.

## 3 XAS and RIXS: Limits of a Purely Atomic Picture

XAS and RIXS provide direct access to the local electronic configuration of the Fe intercalant and represent a natural starting point for understanding the magnetic properties of ${\mathrm{Fe}}_{1/3}{\mathrm{TaS}}_{2}$. In Figure 1e–f,g,h we present polarization‐dependent XAS spectra at the Fe $L_{2,3}$ edges together with grazing and normal incident RIXS measurements, supported by many‐body multiplet cluster calculations and density functional theory calculations (DFT) (see Supplementary Information for further details).

The shape of the XAS and RIXS spectra suggests a 2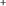 oxidation state of the Fe ion, a claim that is further corroborated by our models. By acquiring the XAS with different polarizations (horizontal LH, vertical LV, see Figure 1e and Figure S2 for the details of the geometry), we observe a linear dichroism (LD) in the Fe $L_{2,3}$ XAS spectra (Figure 1e), signaling a deviation from cubic symmetry. This behavior is consistent with a trigonal distortion of the Fe octahedral environment, as expected from the crystallographic structure of the intercalated compound. The overall line shape and energy position of the absorption edges are well reproduced by multiplet simulations assuming a ${\mathrm{Fe}}^{2+}$ configuration in a $D_{3d}$ point group crystal field, indicating a nominal 3$d^{6}$ electronic filling. The corresponding orbital level scheme is shown in Figure 1f, where the splitting of the 3d manifold reflects the combined effects of crystal field and spin–orbit coupling. RIXS measurements across the Fe $L_{2,3}$ edges further constrain the local electronic structure. The RIXS intensity map (Figure 1g) is dominated by two main loss features at approximately 1 and 3 eV, whose resonance behavior and polarization dependence are characteristic of crystal‐field–allowed d--d excitations. As shown in Figure 1h, these excitations are quantitatively reproduced by the same multiplet model used for the XAS, confirming the internal consistency of the atomic description (the details of the many‐body multiplet cluster model used to model the spectra, sketched in Figure 1f, is provided in Supporting Information). The lower‐energy excitation corresponds predominantly to transitions into the $e_{g}^{\prime }$ manifold, while the higher‐energy loss arises from a dense set of excited states overlapping with the fluorescence background (see also Figure S1 panel c for a visualization of the energy levels). The polarization dependence observed both in XAS and RIXS indicates a ground state characterized by a sizable orbital moment, stabilized by spin–orbit coupling within the $e_{g}^{\prime }$ doublet. This is consistent with previous reports of strong magnetic anisotropy in Fe‐intercalated ${\mathrm{TaS}}_{2}$ compounds [18, 33]. Using the same multiplet parameters, we calculate the local magnetic response and find a large out‐of‐plane anisotropy [18, 34, 37], in agreement with magneto‐optical Kerr effect measurements (Figure 1b). Interestingly, while atomic multiplet description succeeds in reproducing the main spectral features of XAS and the d−d excitations visible in RIXS measurements, important discrepancies emerge when comparing such local picture with experiments. In particular, the calculated saturation magnetization $m_{sat}≃5\;\mu_{B}$ (see Figure S1 panels a and b), that is the result of an electronic configuration with $\left\langle S_{z}\right\rangle ≃4\;\mu_{B}$ and $\left\langle L_{z}\right\rangle ≃1\;\mu_{B}$, exceeds the experimentally measured value $m_{sat}≃4\;\mu_{B}$ [33, 36, 38, 39]. This reduction of the effective magnetic moment indicates that the Fe‐3d electrons are not completely localized and suggests a finite hybridization with the ${\mathrm{TaS}}_{2}$ host lattice. Consistently, the RIXS spectra display well‐defined d−d excitations are observed coexisting with a strong fluorescence contribution, while for some excitation energies a continuum of spectral weight is observed between the elastic peak and the first d−d excitation (Figure 1h and Figure S1d,e). Therefore, while XAS and RIXS remain dominated by local excitations, they carry indirect fingerprints of Fe‐${\mathrm{TaS}}_{2}$ hybridization through the reduced effective magnetic moment. This behavior is representative of an itinerant system with the persistence of localized states typical of intercalated TMDs but it could be also visible in intrinsic metallic magnets as ${\mathrm{Fe}}_{3}{\mathrm{GeTe}}_{2}$ [40] and doped insulating magnets as ${\mathrm{CrGeTe}}_{3}$ [41, 42].

## 4 DFT and ARPES: Evidence for Hybridization

While multiplet calculations correctly capture the local crystal‐field physics of the Fe ions, they do not account for the itinerant electronic degrees of freedom introduced by intercalation. Density functional theory naturally incorporates this itinerancy and allows us to explicitly resolve the hybridization between Fe‐d and Ta‐d states, resulting in a metallic ground state, which is absent in multiplet calculations. DFT calculations on ${\mathrm{Fe}}_{1/3}{\mathrm{TaS}}_{2}$ (see Supporting Information for further details) correctly capture this hybridization, resulting in a $\langle S_{z}\rangle =3.5$ $\mu_{B}$ and $\langle L_{z}\rangle =0.7$ $\mu_{B}$. The reduced saturation magnetization obtained is due to the partial delocalization of the Fe‐d electrons induced by hybridization with Ta‐d states, which transfers spin density away from the Fe site and naturally lowers the local moment.

Inspecting the band structure (see Figure 2a–e for a comparison of the band structure. In Figure 2e the ${\mathrm{Fe}}_{1/3}{\mathrm{TaS}}_{2}$ band structure is projected on Fe‐d orbitals and the colormap correspond to the $S_{z}$ expected value) we note that low dispersive spin‐minority states are located at $E-E_{F}=$–1.8 eV and above $E-E_{F}=$ 1 eV in conduction band. The dispersion along the out‐of‐plane momentum direction is strongly modified by the intercalated ion. In particular, we found a strong Fe‐d‐Ta‐d hybridized spin‐minority states that crosses the Fermi level along the ΓA direction, as highlighted by the red boxes in the Figure 2e. The pronounced dispersion of this hybridized band along $k_{z}$ implies a finite out‐of‐plane hopping amplitude, demonstrating that the corresponding electronic states are intrinsically three‐dimensional rather than confined to individual ${\mathrm{TaS}}_{2}$ layers.

**FIGURE 2:**
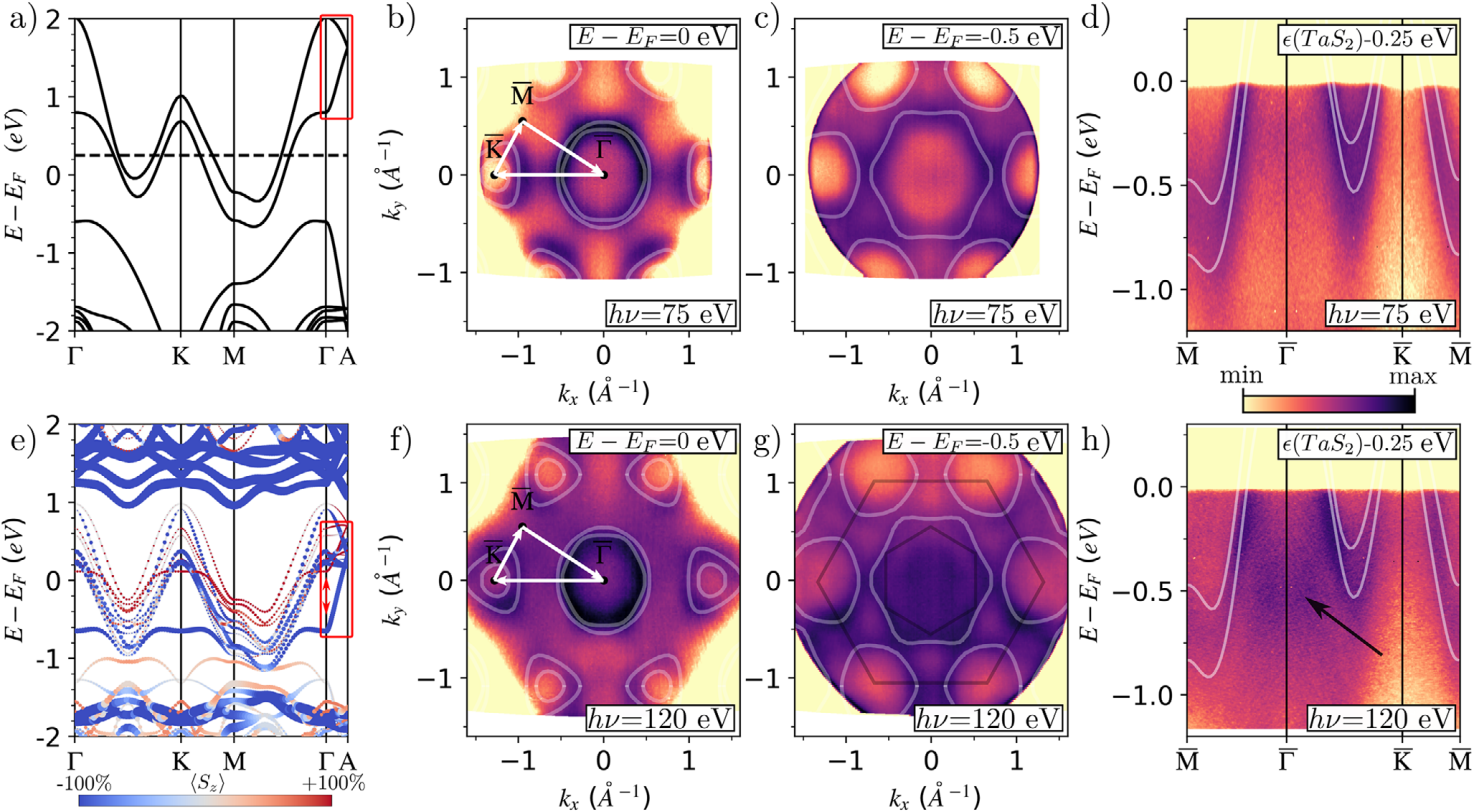
(a, e) Calculated band structures of ${\mathrm{TaS}}_{2}$ and ${\mathrm{Fe}}_{1/3}{\mathrm{TaS}}_{2}$, respectively. In (a), the dashed horizontal line at 0.25 eV indicates the energy shift applied to the ${\mathrm{TaS}}_{2}$ bands to account for electron doping introduced by Fe intercalation (see main text). In (e), the marker size is proportional to the Fe‐d orbital character, while the color scale represents the spin expectation value $\left\langle S_{z}\right\rangle$. (b,f) Fermi surface maps acquired at photon energies hν=75 eV and hν=120 eV, respectively. The indicated high‐symmetry directions define the momentum path used for the band structure analysis. (c, g) Constant‐energy maps measured at $E-E_{F}=-0.5$ eV for hν=75 eV and hν=120 eV, respectively. (d, h) Experimental band dispersions extracted along the momentum paths indicated in (b) and (f). The calculated ${\mathrm{TaS}}_{2}$ band structure obtained using the HSE06 functional, rigidly shifted by 0.25 eV to account for Fe‐induced electron doping, is overlaid for comparison. Black arrows highlight an additional band that is absent in pristine ${\mathrm{TaS}}_{2}$.

Indeed, we found that the Fe ions induce a small antiferromagnetically coupled moment on the nearest Ta atoms (∼ –0.04 $\mu_{B}$). Moreover, analyzing the occupation matrix in our first principle calculations (see Supporting Information for further details) we found that the glide mirror symmetry of the host compound imposes a different local orientation of the Fe in‐plane orbitals. This peculiar property of intercalated TMD is ultimately the origin of the emergence of altermagnetic phases in this class of compounds when A‐type antiferromagnetism is present [21, 22, 23, 24]. Therefore, our first‐principle calculations show that beyond the presence of localized Fe‐d states, responsible for the magnetic properties of the compound, the out‐of‐plane energy dispersion is significantly modified by the ion intercalation, resulting in an additional dispersive band crossing the Fermi level.

Evidence for Fe‐d–Ta‐d hybridization was obtained from ARPES measurements, shown in Figure 2. In particular, we report ARPES spectra acquired with photon energy hν = 75 eV in panels b–d and hν = 120 eV in panels f–h at T = 15 K. We superimpose DFT calculation of the pristine 2H‐${\mathrm{TaS}}_{2}$ along the MΓKM path shifted by 0.25 eV to account for electron doping induced by the TM intercalant atom and better compare with the electronic structure of the host compound (the $k_{z}$ dispersion mainly affect the broadening in the M point [43]). At hν = 75 eV, see Figure 2b,c, the isoenergy maps along the plane $\bar{\Gamma }\bar{K}\bar{M}$ highlight the presence of a hole pocket at $\bar{K}$ similar to the one present in 2H‐${\mathrm{TaS}}_{2}$ [43, 44, 45].

In Figure 2d we report the band structure along the $\bar{M}\bar{\Gamma }\bar{K}\bar{M}$ lines of the surface BZ, showing Ta−d bands crossing the Fermi level and no significant difference with respect to pristine 2H‐${\mathrm{TaS}}_{2}$ apart from a substantial rigid doping and diffuse spectral weight at $\bar{\Gamma }$. Changing the photon energy, hν = 120 eV, novel features are visible. Indeed, the energy cut at $E-E_{F}$ = ‐0.5 eV show signal near the BZ center, where no bands of pristine 2H‐${\mathrm{TaS}}_{2}$ are present [43]. These bands are visible in Figure 2h where the band structure along the $\bar{M}\bar{\Gamma }\bar{K}\bar{M}$ is reported. Our first principle calculations on ${\mathrm{Fe}}_{1/3}{\mathrm{TaS}}_{2}$ show that these bands are hybridized Fe‐d and Ta‐d bands (see Figure 2e) dispersing in the $\bar{\Gamma }\bar{K}\bar{M}$ plane.

For improving the comparison of DFT calculations with experimental measurement we report in Figure 3 high‐statistics measurement along ΓK parallel direction compared with intensity simulation taking into account interference effects from initial wavefunction (see Supporting Information for further detail). We further show the second derivative of the intensity in the left panel of Figure 3a to better resolve the splitting of the bands. In the left panel of Figure 3b we report band structure of the compound in the ${\mathrm{Fe}}_{1/3}{\mathrm{TaS}}_{2}$ unit cell.

**FIGURE 3:**
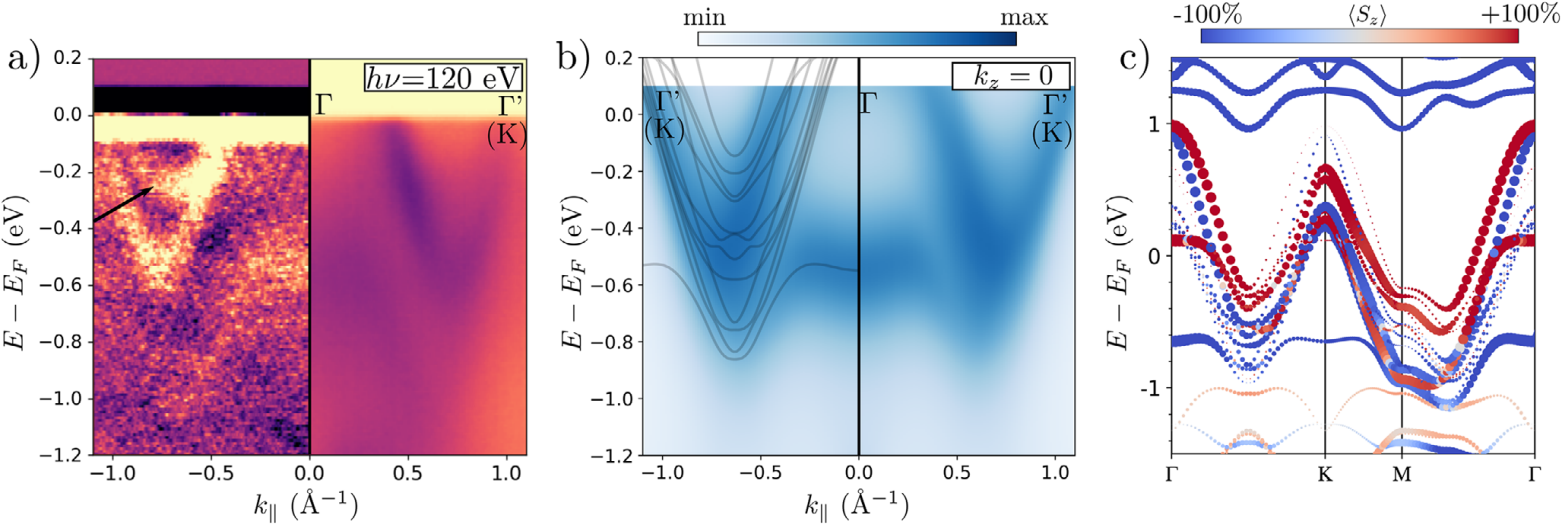
(a) Energy dispersion along the Γ−K line acquired at hν=120 eV. The left panel report the second derivative of the right panel plot. (b) Simulation of the ARPES intensity (See Methods for further details). On the left, we superimposed the band structure calculated using the PBE+U+SOC functional, shifted by 0.1 eV to account for an overall p‐doping in the sample. (c) Unfolded and spin‐polarized band structure. The size of the points is proportional to the weight of the unfolding, while the colormap is for the spin polarization $\left\langle S_{z}\right\rangle$.

We note that Ta‐d bands that in 2H‐${\mathrm{TaS}}_{2}$ were completely empty by charge density wave distortion [43, 44, 46] are now filled and reach $E-E_{F}∼$ –0.3 eV (as highlighted by black arrow in the Figure 3a). The observed band broadening with respect to pristine 2H‐${\mathrm{TaS}}_{2}$ is attributed to Fe intercalation, which introduces hybridization between Fe‐d and Ta‐d states and local distortion redistributing the spectral weight of the folded Ta‐d bands. This feature is correctly captured by our intensity simulation as shown in Figure 3b,c. Furthermore, our simulations fully describe the vanishing spectral weight observed in the experiment of the Fe‐d derived band in ${\bar{\Gamma }}^{\prime }$ of the $\sqrt{3}\times \sqrt{3}R30^{\circ }$ BZ as shown in Figure 3b. In these simulations, the initial state in the matrix element M is linear combination of Fe‐$d_{z^{2}}$, Fe‐$d_{x^{2}-y^{2}}$, Fe‐$d_{xz}$ and Ta‐$d_{z^{2}}$, Ta‐$d_{x^{2}-y^{2}}$, Ta‐$d_{xz}$ accounting for the LH polarization of the light. The breaking of time reversal symmetry and centrosymmetry by intercalation of ferromagnetic aligned Fe ions naturally leads to analyze the spin splitting of the measured bands. In Figure 3c and in Figure S7, we show the unfolded band structure (from the $\sqrt{3}\times \sqrt{3}R30^{\circ }$ unit cell to the 2H‐${\mathrm{TaS}}_{2}$ unit cell) at different $k_{z}$, where the colormap represents the spin polarization along the z axis ($\left\langle S_{z}\right\rangle$). The spin degeneracy that, in the pristine system, arises from the compensation of Ising spin splitting due to glide mirror symmetry between layers is now lifted. The hybridization between the Fe local magnetic moments and the Ta conduction states induces a pronounced spin splitting of the Ta‐d bands, effectively acting as a Zeeman field along z on otherwise spin‐degenerate states. This hybridization arises from a Kondo‐like Hamiltonian

(1) $$H_{dd}=J_{dd}\sum_{i}S_{i}\cdot s\left(r_{i}\right)$$

where the itinerant electrons (${\mathrm{Ta}}_{d_{z}^{2}}$ like) with spin‐density **s**($r_{i}$) interact with localized Fe‐d spins $S_{i}$. Thus, the induced spin splitting of the Ta‐derived bands reflects an effective exchange field, $\Delta_{ex}∼J_{dd}$, generated by the Fe moments $S_{i}$, indicating that the itinerant electrons actively participate in the magnetic coupling, with a strength $J_{dd}$.

The splitting is significantly larger in the ΓKM plane than in the AHL plane; for instance, at the Γ point the spin‐minority band is occupied while the spin‐majority band remains unoccupied, enabling the observation of this spin‐polarized band at the Γ point in the ARPES spectra, as clearly visible in Figure 3a,b. Interestingly, as demonstrated by high‐temperature XAS and RIXS measurements (Figure 1e–g,h), the Fe atoms retain well‐defined local excitations even in the absence of long‐range magnetic order. This observation supports the persistence of exchange splitting above the magnetic transition temperature reported in similar intercalated compounds [47] (see Supporting Information section “Spin splitting above the Curie temperature”).

Taken together, DFT and ARPES demonstrate that Fe intercalation generates a spin‐polarized, $k_{z}$‐dispersive itinerant band that qualitatively alters the electronic dimensionality of the system, manifesting as a previously overlooked spin‐polarized band [18, 48], and provides the microscopic basis for interlayer magnetic coupling.

The presence of such hybridized states, can, in principle, mediate the coupling between different 2D magnetic Fe lattices, changing the magnetic behavior from 2D‐like to 3D. Thus, to probe the 3D dispersion along the out‐of‐plane momentum direction we carried out soft‐X‐rays ARPES (SX‐ARPES) with photon energies from 350 eV to 700 eV and circular right (CR) polarization, reporting the corresponding spectra in Figure 4 (see Supporting Information for further detail where we report ARPES spectra acquired with circular right (CL) polarization). In Figure 4a,b we show SX‐ARPES Fermi surface map in the ΓAK plane and the corresponding first‐principles DFT simulation of the spectrum.

**FIGURE 4:**
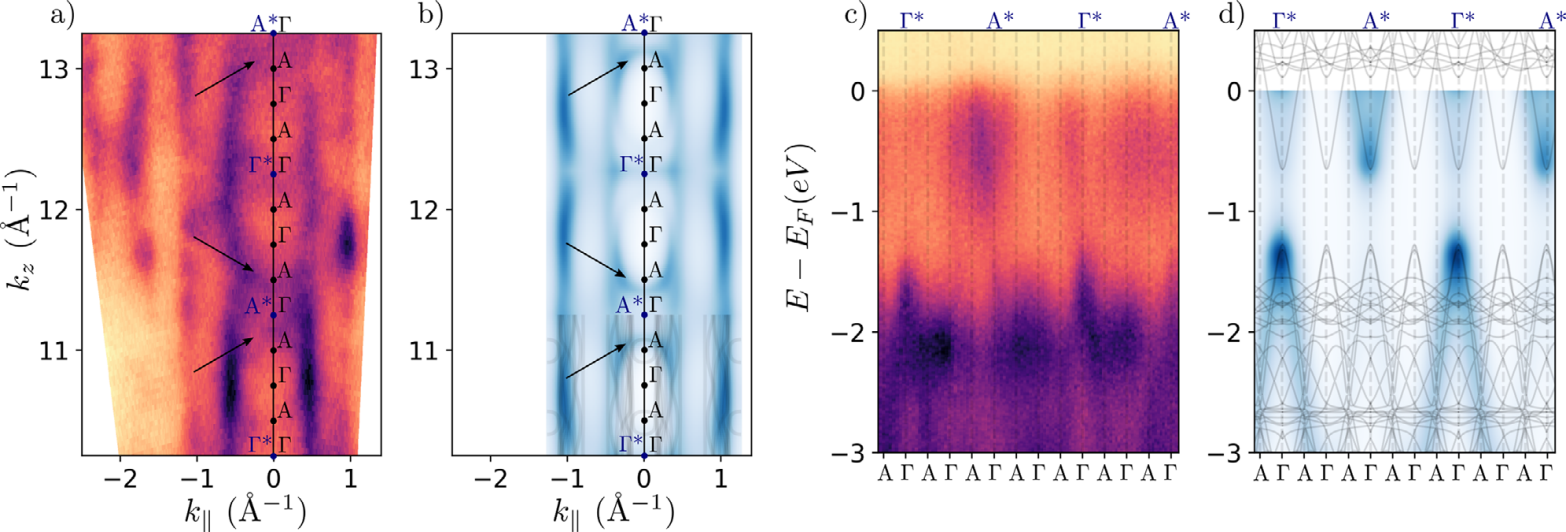
(a) Soft X‐rays ARPES Fermi surface map in the ΓAK plane using CR polarized light. We reported in black several BZ along the $k_{z}$ momentum. In dark blue we reported symmetry points stemming for the observed periodicity in the ARPES spectrum. The black arrows indicate the Fermi surface lines not present in the ${\mathrm{TaS}}_{2}$ spectrum. (b) ARPES intensity simulation of the ΓAK plane. For the intensity simulations we used Fe‐d and Ta‐d complex projections. In the bottom, we reported the Fermi surface as seen from the bare band structure. (c) Band dispersion along the ΓA direction. (d) ARPES intensity simulation of the ΓA direction. In black, we superimpose $k_{z}$ dispersion of the band at $\left(k_{x},k_{y}\right)=\left(0,0\right)$.

The first important evidence is the presence of a three‐dimensional Fermi surface topology, showing closed 3D sheets, as evidenced by the pronounced features indicated by the black arrows in Figure 4a,b. On the contrary, these features are completely absent in the host compound [43] (see also Figure S8 for a comparison of the measured out‐of‐plane band dispersion in ${\mathrm{Fe}}_{1/3}{\mathrm{TaS}}_{2}$ and calculations in ${\mathrm{TaS}}_{2}$), which shows a 2D cylindrical Fermi surface. Our first‐principle calculation, see Figure 4b, confirms that these states originate from the out‐of‐plane hybridization between Ta‐$d_{z^{2}}$ and Fe‐$d_{z^{2}}$, which cross the Fermi level along the Γ−A direction with a linear dispersion (see Figure 4d), while the dispersion of this band along the in‐plane momentum shows a nearly zero velocity at Γ suggesting a pronounced electronic anisotropic transport behavior (see Figures S5 and S6 where we report dispersion in the ΓK with different photon energy and $k_{z}s$ going from Γ to A). To properly describe such band, we first note that the measured Fermi surface map shows a $k_{z}$ periodicity four times larger than the one of the lattice of ${\mathrm{Fe}}_{1/3}{\mathrm{TaS}}_{2}$ (we introduced the $\Gamma^{*}$ and $A^{*}$ points to describe the observed periodicity). These multiple doublings of the signal cannot be captured by band structure calculations, reported in the bottom of Figure 4b, which by definition has the periodicity of the BZ unit cell. We ascribe this experimental evidence to a consequence of quantum interference effects between atoms belonging to different layers combined with the geometric effect of intercalant Fe ion. These interference effects have been already observed in pristine 2H‐${\mathrm{TaS}}_{2}$ [43] and 2H‐${\mathrm{NbS}}_{2}$ [49] and arise from the phase mismatching in different sublattice sites of the initial state wavefunction (different TMD layers). In the latter cases, the period is doubled. In this case, the already doubled period is doubled again due to the intercalation of Fe ions in the vdW gap of 2H‐${\mathrm{TaS}}_{2}$ that induces an additional contribution to the initial Bloch wavefunction's phase, whose impact on the intensity of the SX‐ARPES signal can be measured. Indeed, the photoemission matrix element can be written as $M∼\left\langle \psi_{f}\right|\left(A\cdot p\right)\sum_{j}C_{j}^{nk}|\phi_{j}\rangle$, where j stand for sublattice indices, $C_{j}^{nk}=\left\langle \phi_{j}|\psi_{kn}^{KS}\right\rangle$ is the projection of the Kohn–Sham wavefunction on suitable atomic orbitals $\left\langle \phi_{j}\right|$. This demonstrates that ARPES intensity is proportional to the linear combination of sublattice projected initial state wavefunction. To better clarify the origin of this pure quantum effect induced by the Fe intercalants, we construct one dimensional (1D) models to capture the out‐of‐plane dispersion in the pristine and intercalated phases (see Supporting Information and Figure S3). While the $k_{z}$ dispersion in pristine 2H‐${\mathrm{TaS}}_{2}$ is well captured by a monoatomic chain model, ${\mathrm{Fe}}_{1/3}{\mathrm{TaS}}_{2}$ requires two inequivalent atomic sites. Nonmagnetic band‐structure calculations (see Figure S3) and relative 1D model elucidate the underlying mechanism: at the $\Gamma_{1}$ point, the wavefunctions on sublattices A and B acquire a relative π‐phase shift, in contrast to $\Gamma_{0}$ and $\Gamma_{2}$, where they remain in phase. The same behavior is observed in both measured and simulated ARPES spectra, where the spectral weight at $\Gamma^{*}$ differs from that at $A^{*}$ due to phase mismatch in the initial‐state wavefunctions induced by the intercalant atoms. Therefore, by comparing SX‐ARPES and first‐principle‐calculation we unveil the out‐of‐plane dispersion of previously overlooked band induced by the Fe intercalant atom in the vdW gap of ${\mathrm{TaS}}_{2}$. Moreover, apart from the methodology we used to reconstruct the signal, it is important to note that such strong interference effects is reflecting once more the relevance of the Fe intercalation in shaping the band structure of the material (see also Figure S8 for a comparison with the $k_{z}$ dispersion of the pristine 2H‐${\mathrm{TaS}}_{2}$).

## 5 The Role of Interlayer Coupling

To understand the role of this band in shaping the magnetic properties of the material, we calculated the interlayer magnetic coupling by total energy difference method as a function of the out‐of‐plane lattice parameter c. Interestingly, the calculated value for the interlayer coupling is one order of magnitude larger than the one calculated for paradigmatic bulk 2D magnet ${\mathrm{CrI}}_{3}$ [50]. We also stabilize a higher‐energy phase with $\left\langle L_{z}\right\rangle ≃0\;\mu_{B}$ by explicitly controlling orbital occupancy [51, 52, 53] and mixing parameter in the self‐consistent cycle; in this case, the interlayer coupling changes sign, yielding an antiferromagnetic ground state. This underscores the necessity of stabilizing the correct orbital configuration to properly capture the magnetic ground state, which is otherwise missed when the orbital degree of freedom is not accounted for [54].

The magnetic coupling between Fe atoms in each layer are effectively coupled through the dispersive out‐of‐plane band, in an anisotropic RKKY‐like mechanism as described by Equation (1).

As a result, the effective interlayer magnetic coupling $J_{z}$ depends on the spin susceptibility $\chi^{\alpha }$ and scales as $J_{z}^{\alpha }∼J_{dd}^{2}\chi^{\alpha }$, where $J_{dd}$ is the intercalant–electron exchange coupling and α denotes the spin anisotropy channel. In momentum space, the out‐of‐plane component of the susceptibility can be written as

(2) $$\chi^{\alpha }\left(q_{z}\right)=\sum_{k}\frac{f\left(\varepsilon_{k}\right)-f\left(\varepsilon_{k+q_{z}}\right)}{\varepsilon_{k+q_{z}}-\varepsilon_{k}}{\left|M_{S^{\alpha }}\left(k,k+q_{z}\right)\right|}^{2},$$

where $f(\varepsilon_{k})$ is the Fermi–Dirac distribution and $M_{S^{\alpha }}\left(k,k+q_{z}\right)=\langle k+q_{z}|S^{\alpha }\left|k\right\rangle$ denotes the spin matrix element. In the absence of $k_{z}$ dispersion, the electronic structure is effectively decoupled along the out‐of‐plane direction, such that $\varepsilon_{k}$ shows negligible dependence on $k_{z}$. In this limit, the susceptibility exhibits only a weak dependence on $q_{z}$, reflecting the reduced coherence of itinerant carriers along the interlayer direction, corresponding to a strongly localized real‐space interlayer response with a short effective spatial range. In contrast, the presence of a finite $k_{z}$ dispersion introduces a non‐zero band curvature along the out‐of‐plane direction, increasing the phase space that contribute to interlayer coupling and thereby enhancing $\chi^{\alpha }\left(q_{z}\right)$. This effect strengthens the interlayer RKKY interaction by enabling coherent itinerant electronic propagation along the c axis. The resulting coupling increases with the band curvature, i.e. with the effective interlayer hopping amplitude $t^{*}$. Indeed, we estimate the effective hopping $t^{*}$ by expanding near the Γ point the first‐principle energy dispersion $\varepsilon \left(k_{z}\right)=a_{1}+t^{*}c^{2}k_{z}^{2}$. In Figure 5 we show the evolution of these quantities as a function of the out‐of‐plane lattice parameter c. Reducing c systematically enhances the interlayer exchange $J_{z}$, revealing the sensitivity of the magnetic coupling to the interlayer spacing. In parallel, the effective hopping $t^{*}$ of the out‐of‐plane dispersive band exhibits an increase. Moreover, the antiferromagnetically aligned moment on the otherwise non‐magnetic Ta site grows in absolute value with increasing $J_{z}$, further indicating that the interlayer exchange is mediated by hybridization Fe‐d‐Ta‐d bands.

**FIGURE 5:**
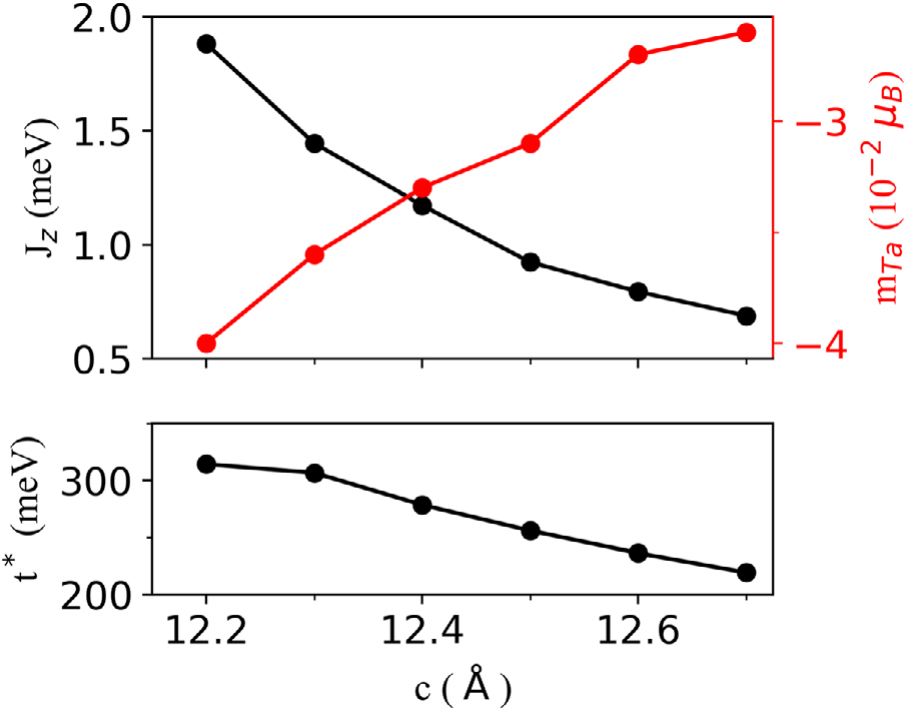
Magnetic interlayer coupling $J_{z}$ and estimated effective hopping $t^{*}$ of the out‐of‐plane dispersive band crossing the Fermi level as a function of the lattice parameter c.

Therefore the emergence of a $k_{z}$ dispersive Fe–Ta hybridized band provides the microscopic origin of the interlayer exchange, with $J_{z}$ increasing with the out‐of‐plane hopping amplitude. Reducing the interlayer spacing enhances the out‐of‐plane hopping of the itinerant band, which in turn strengthens the interlayer magnetic exchange. This concurrence demonstrates that the hybridized itinerant electrons contributing to the $k_{z}$‐dispersive band provide a channel for mediating $J_{z}$, establishing a direct and tunable link between lattice geometry, electronic dispersion, and interlayer magnetic exchange.

## 6 Conclusion

In conclusion, we have shown that magnetic intercalation in metallic van der Waals materials qualitatively reshapes the electronic structure by generating itinerant, $k_{z}$ dispersive states that fundamentally alter the nature of magnetic coupling. Our analysis demonstrates that a purely atomic picture cannot account for the magnetic behavior of this compound. Instead, we reveal the essential role of hybridization between the magnetic Fe intercalants and the metallic ${\mathrm{TaS}}_{2}$ host, manifested in a strongly three‐dimensional dispersive band and a reduced magnetization at saturation.

The emergence of a spin‐polarized Fe–Ta hybridized band crossing the Fermi level provides an efficient itinerant channel for interlayer spin propagation. This establishes a direct microscopic link between the electronic dispersion and three‐dimensional magnetic order, enabling a tunable out‐of‐plane exchange interaction that ultimately gives rise to A‐type antiferromagnetism in intercalated altermagnets [21, 22, 23, 24], as well as to a tunable helical spin texture [13]. These findings uncover the microscopic mechanism behind the divergence from purely two‐dimensional magnetism in this system and provide a foundation for understanding and engineer the emergence of exotic magnetic phases in transition metal‐intercalated dichalcogenides.

## Conflicts of Interest

The authors declare no conflicts of interest.

## Supporting information




**Supporting File**: advs76533‐sup‐0001‐SuppMat.pdf.

## Data Availability

The data that support the findings of this study are available from the corresponding author upon reasonable request.
