# Supplementary material for: Emergent 3D Fermiology and Magnetism in an Intercalated Van der Waals System
Source: Adv Sci (Weinh). 2026 Jul 27:e76533. Online ahead of print. doi: 10.1002/advs.76533 (PMC13403743; doi:10.1002/advs.76533)
Supplement: Supplementary file 1 — Supporting File: advs76533‐sup‐0001‐SuppMat.pdf. [file ADVS-9999-e76533-s001.pdf]

Supplemental material and supporting information for

# Emergent 3D Fermiology and Magnetism in an Intercalated Van der Waals System

Luigi Camerano[a,b], Emanuel A. Martínez [b], Victor Porée[c,d], Laura Martella [a], Dario Mastrippolito [e], Debora Pierucci [e], Franco D'Orazio [a], Polina M. Sheverdyaeva [f], Paolo Moras [f], Enrico Della Valle [g], Tianlun Yu [g], Moritz Hoesch [h], Craig M. Polley [i], Thiagarajan Balasubramanian [i], Alessandro Nicolaou [d], Luca Ottaviano [a,b], Vladimir N. Strocov [g], Gianni Profeta [a,b], Federico Bisti [a]

[a] Department of Physical and Chemical Sciences, University of L'Aquila, Via Vetoio 67100 L'Aquila, Italy
[b] CNR-SPIN L'Aquila, Via Vetoio, 67100 L'Aquila, Italy c/o Department of Physical and Chemical Sciences, University of L'Aquila, Via Vetoio, 67100 L'Aquila, Italy
[c] Univ Rennes, CNRS, Institut des Sciences Chimiques de Rennes-UMR6226, 35042 Rennes, France
[d] Synchrotron SOLEIL, L'Orme des Merisiers, Saint-Aubin, BP 48, F-91192 Gif-sur-Yvette, France
[e] Sorbonne Université, CNRS, Institut des NanoSciences de Paris, 4 place Jussieu, 75005, Paris, France
[f] CNR-Istituto di Struttura della Materia (CNR-ISM), Strada Statale 14, km 163.5, 34149 Trieste, Italy
[g] Swiss Light Source, Paul Scherrer Institute, CH-5232 Villigen PSI, Switzerland.
[h] Deutsches Elektronen-Synchrotron DESY, Notkestrasse 85, 22607 Hamburg, Germany
[i] MAX IV Laboratory, Lund University, Lund, Sweden

## CONTENTS

I. Theoretical simulations 2

II. Experimental methods 2
A. MOKE methods 2
B. XAS-RIXS methods 3
C. ARPES and SX-ARPES 4

III. Models for $k_z$ dispersion 5

IV. Spin splitting above the Curie temperature 6

V. Complementary data and simulations 7

References 11

## I. THEORETICAL SIMULATIONS

Density functional theory calculations were performed using the Vienna ab-initio Simulation Package (VASP) [1, 2], using the generalized gradient approximation (GGA) in the Perdew-Burke-Ernzerhof (PBE) parametrization for the exchange-correlation functional [3], including SOC. Interactions between electrons and nuclei were described using the projector-augmented wave method. Energy thresholds for the self-consistent calculation was set to $10^{-6}$ eV and force threshold for geometry optimization $10^{-4}$ eV $\AA^{-1}$. For the calculation of interlayer magnetic exchange the energy thresholds for the self-consistent calculation was set to $10^{-7}$. A plane-wave kinetic energy cutoff of 550 eV was employed. The Brillouin zone was sampled using an $8 \times 8 \times 4$ Gamma-centered Monkhorst-Pack grid. To account for the on-site electron-electron correlation on localized Fe-$d$ orbitals we used the GGA+U approach with an effective Hubbard term $U = 4.42$ eV as we calculated with linear response theory [4]. The 2H-$TaS_2$ lattice parameter are set to the experimental ones: $a = 3.31$ Å and $c = 12.07$ Å [5], while for the $Fe_{1/3}TaS_2$ $a = 5.737$ Å and $c = 12.28$ Å [6, 7].

For the band structure of 2H-$TaS_2$ in Fig. 3 a-d-h we used HSE06 functional [8–10] to better describe bands around the M point [11], using the FHI-AIMS simulation package [12, 13], which is an accurate all-electron full-potential electronic structure package based on numeric atom-centered orbitals, with so-called "tight" computational settings. The screened hybrid functional HSE06 with the mixing factor $\alpha = 0.25$ and screening parameter $\omega = 0.11$ $\text{Bohr}^{-1}$ was used for the exchange-correlation energy.
Due to the localization of Fe-$d$ orbitals, different metastable phases can be stabilized for $Fe_{1/3}TaS_2$. To stabilize large orbital moment phase we used a mixing parameter $\alpha_{mix} = 0.22$ and the occupation matrix control as implemented in VASP [14]. We notice that this phase is indeed the ground state of the system by comparing the total energy.
ARPES intensity simulations are based on the standard model [15–17], where the Lorentzian spectral function is weighted with the square modulus of the matrix element:

$$I(\mathbf{k}, E) = \frac{\sigma}{(E - E_{\mathbf{k}})^2 + \sigma^2} |M|^2 , \quad (1)$$

where $E_{\mathbf{k}}$ are the first principle computed eigenvalues, $\sigma$ is the spectral width. $|M|$ is an adapted version of the general $M = \langle \psi_f | \mathbf{A} \cdot \mathbf{p} | \psi_i \rangle$ in a DFT framework, that takes into account interference from initial state wavefunction as $M \sim \sum_j \pm C_j^{n\mathbf{k}}$, where $j$ stand for sublattice indices, $C_j^{n\mathbf{k}} = \langle Y_j | \psi_{\mathbf{k}n}^{KS} \rangle$ is the projection of the Khon-Sham wavefunction on suitable atomic orbitals $\langle Y_j |$ and $\pm$ fix the symmetry of the initial state according to the polarization of the light [11, 18, 19]. The coefficients $C_j^{n\mathbf{k}}$ contains the information about interference effects.
The relative rotation of the Fe-$d$ orbitals due to the glide mirror symmetry is analyzed using the $d$ density matrix for the down spinon component $n^{\downarrow}_{Fe-d}$. Defining $U$ as the rotation matrix for the cubic harmonics we can write $n^{\downarrow}_{Fe_1-d} = U(\phi = 60^\circ)\, n^{\downarrow}_{Fe_2-d} U^{\dagger}(\phi = 60^\circ)$.
The quantum many body script language Quanty [20–22] was used to reproduce the XAS and RIXS spectra considering a crystal electric field model. The local reference frame used to express the incident and outgoing polarization is represented in Fig. S2b. Slaters integrals, $F_{2,4}$ and $G_{1,3}$ were rescaled to 75 % and 65 % of their Hartree-Fock values, respectively. The spin-orbit coupling was also scaled down by 50 %. In order to account for the linear dichroism observed in the absorption spectra, the $D_{3d}$ point group was considered to capture the crystal-electric field experienced by the $Fe^{2+}$ ions. In this point group, the five $3d$ orbitals split into 3 levels : one non-degenerate ($a_{1g}$) and two doubly degenerated ($e_g$ and $e'_g$) orbitals. Based on the DFT calculations and observed dd transitions, the following orbital energies were used: $e'_g$ = -0.2 eV, $a_{1g}$ = 0 eV, $e_g$ = 0.7 eV. A spectral broadening of 0.4 eV is added to compare with RIXS experimental measurement.

## II. EXPERIMENTAL METHODS

### A. MOKE methods

MOKE measurements were performed with an homemade experimental setup using laser with 625 nm wavelength. MOKE rotation was measured as a function of the out-of-plane magnetic field (aligned with the crystallographic $c$ axis), in the range ±5600 Oe. The bulk samples were cleaved before the MOKE measurements, which were carried out in a cryostat at a base pressure of $1 \times 10^{-6}$ mbar.

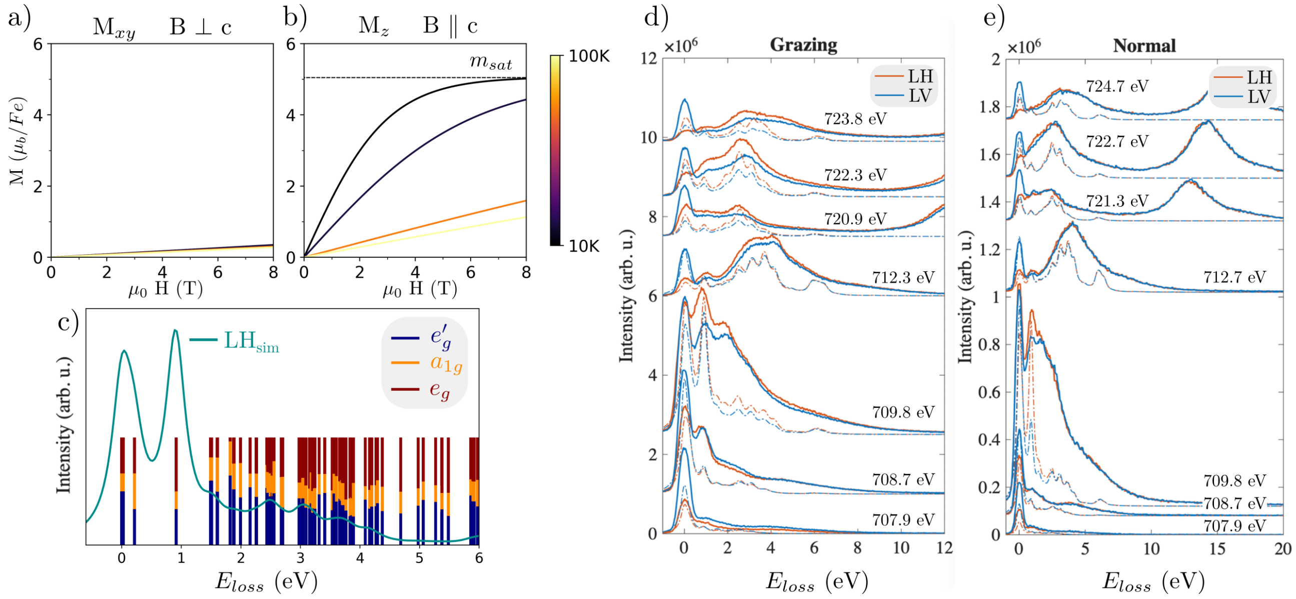


FIG. S1. Magnetization curves with field perpendicular in a) and parallel in b) to the crystallographic $c$ axis showing the role of anisotropy. c) Calculated many-body energy levels superimposed on a RIXS spectrum. The colour of each level indicates the occupation of the Fe 3d orbitals. d) and e) are additional RIXS spectra and simulation thereof in grazing and normal geometry.

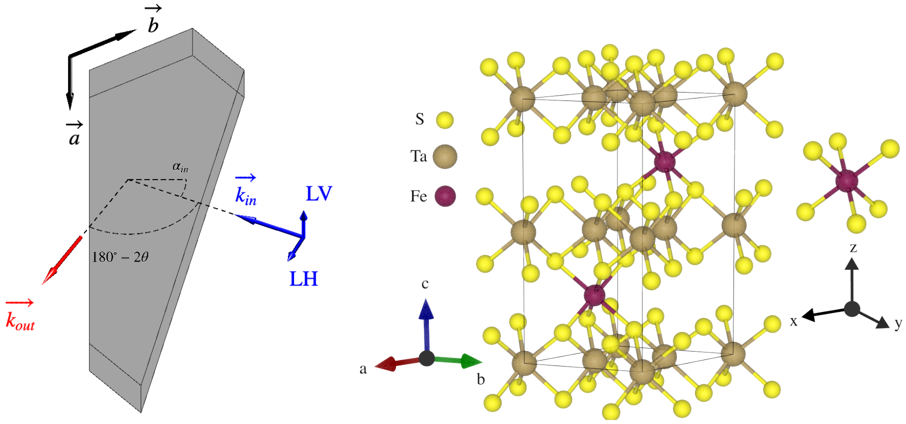


FIG. S2. a) Scheme of the experimental geometry presenting the orientation of the incident and outgoing radiation with respect to the crystal and its crystallographic axes. b) Crystallographic unit cell of $Fe_{1/3}TaS_2$ with Fe, Ta and S atoms depicted in violet, khaki and yellow, respectively. The local xyz reference frame used for the model calculation is also included, having its x and z axes collinear with the crystallographic a and c directions.

### B. XAS-RIXS methods

X-ray absorption spectroscopy and resonant inelastic x-ray scattering measurements at the Fe $L_{2,3}$ edges were performed on the inelastic branch of the SEXTANTS beamline of the synchrotron SOLEIL [23]. RIXS spectra were acquired employing the AERHA spectrometer [24] with a fixed scattering angle of $2\theta = 85°$ and an overall resolution of 570 meV, while XAS spectra were obtained via total electron yield with a resolution of 120 meV. All measurements were carried out at room temperature on a freshly cleaved millimeter-size single crystal using the MAGELEC sample environment [25]. The latter was glued to a copper Omicron-type sample holder using silver paint, with its $a$ crystallographic axis along the vertical experimental direction (i.e. parallel to the vertical beam polarisation LV) and its $c$ crystallographic axis within the scattering plane (i.e. the horizontal experimental plane). XAS and RIXS spectra were acquired using linear (vertical LH, horizontal LV) polarisation in both grazing and normal geometry (with incident angle of 20° (Q=0,-0.1860,-0.4492) and 80° (Q=0,0.2960,-0.3857), respectively). The 20° RIXS data are depicted in Fig. S1d, while the 80° data are shown in Fig. S1e. A scheme of the experimental

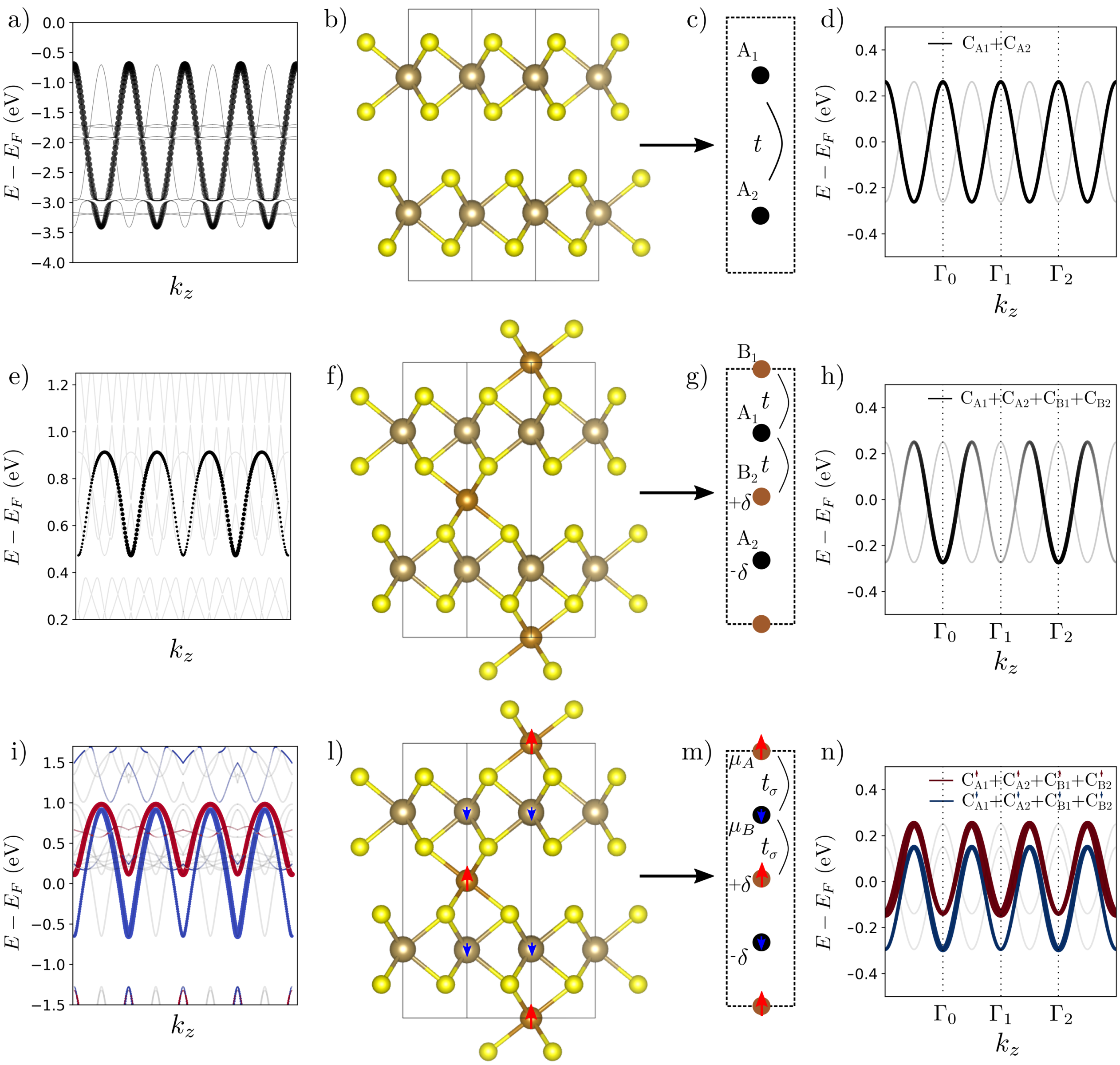


FIG. S3. a)-e)-i) First principle out-of-plane dispersion of pristine 2H-$TaS_2$, non-magnetic $Fe_{1/3}TaS_2$ and ferromagnetic $Fe_{1/3}TaS_2$, respectively. b)-f)-l) Magnetic and structural unit cell of the corresponding phases. c)-g)-m) Sketch of one dimensional models capturing the $k_z$ dispersion. d)-h)-n) Band structure of the one dimensional model.

geometry is given in Fig. S2a. Additionally, a RIXS map covering the Fe $L_{2,3}$ energy range was collected in grazing incidence, using a circularly polarised incident radiation.

## C. ARPES and SX-ARPES

ARPES experiments were performed at the Bloch beamline of the MAX IV synchrotron (Sweden) using DA30-L ScientaOmicron analyzer with a horizontal slit. The photon energies used were in the 75 and 130 eV range with linear horizontal polarized light (LH polarization) and the sample temperature was T = 15 K. The $k_z$ dispersion is calculated using an inner potential of 10 eV. The samples, commercial crystals from HQ graphene, were exfoliated in the UHV chamber using a Kapton tape. Preliminary ARPES data were also collected at VUV-beamline of Elettra

synchrotron (Trieste, Italy) using a Scienta R4000-WAL analyzers with a horizontal slit. These latter data are not reported in the present study for the sake of conciseness. SX-ARPES experiments were performed at the P04 beamline of the PETRAIII synchrotron at DESY (Germany) delivering circular polarized light. The endstation, migrated from the ADRESS beamline of the Swiss Light Source and based on a grazing-incidence experimental geometry, used a PHOIBOS225 analyzer with a vertically oriented slit. The measurements used photon energies in the range 350-700 eV. The combined energy resolution varied between 50 and 100 eV, respectively. The sample temperature was T = 15 K. Low energy electron diffraction (LEED) showed the $\sqrt{3}\times\sqrt{3}R30^{\circ}$ pattern with respect to the pristine 2H-$TaS_2$ typical of the $x = 1/3$ concentration (see Fig. 1c).

## III. MODELS FOR $k_z$ DISPERSION

In order to describe the $k_z$ dispersion of 2H-$TaS_2$ and intercalated $Fe_{1/3}TaS_2$ we report in Fig. S3 first principle calculation and one dimensional models capturing its main features. In Fig.S3 panel a we report first-principle calculation where the size of the points is proportional to the square modulus of $M \sim \sum_{S_j} C^{n\mathbf{k}}_{S_j}$ as in Ref. [11], where $S_j$ is S-$p_z$ orbitals. This band dispersion can be described by one dimensional monoatomic chain as shown in Fig. S3 panel d where the weight is proportional to the sum of A1 and A2 sites. First-principle calculation of non-magnetic $Fe_{1/3}TaS_2$ (Fig. S3 panel e) show that the weight, proportional to the square modulus of $M \sim \sum_{Ta_j} C^{n\mathbf{k}}_{Ta_j} + \sum_{Fe_j} C^{n\mathbf{k}}_{Fe_j}$, shows a different modulation along $k_z$ with respect to the pristine case (where $Ta_j$ and $Fe_j$ represent Ta-$d^{z^2}$ and Fe-$d^{z^2}$ orbitals). This feature is well captured by a monoatomic chain with two atoms with different masses (see sketch in Fig. S3 panel g). The band structure weighted with $|C_{A1} + C_{A2} + C_{B1} + C_{B2}|^2$ well describe the $k_z$ modulation, demonstrating that it arises from a pure geometric contribution to the Bloch phase. Finally, to describe the dispersion of the realistic ferromagnetic $Fe_{1/3}TaS_2$ we constructed a spinful tight-binding model with different Zeeman $\mu_i$ $(i = A, B)$, on-sites $(\pm\delta)$ (see Fig. S3 panel i-l-m-n) and spin dependent hoppings $t_\sigma = \sigma t$ with $\sigma = \pm 1$.

## IV. SPIN SPLITTING ABOVE THE CURIE TEMPERATURE

In magnetic materials, the disappearance of long-range ferromagnetic order at $T_C$ does not necessarily imply the disappearance of local magnetic moments or exchange fields. Instead, fluctuating local moments may persist above $T_C$, leading primarily to spectral broadening and redistribution of spectral weight rather than to a simple closure of the exchange splitting expected within a rigid-band or mean-field picture. This behavior closely resemble observation of persistent splitting above magnetic transition temperature in $Cr_{1/3}NbS_2$ [26], where temperature-dependent ARPES measurements show that exchange splitting persists above $T_c$, consistent with the survival of short-range magnetic correlations and local moments. In our case, the persistence of localized Fe moments even at room temperature is supported by the RIXS measurements, which retain the characteristic atomic multiplet features of Fe ions. To further clarify this point, we constructed a simple one-dimensional tight-binding model including local magnetic moments without long-range order. The Hamiltonian is given by

$$H = t \sum_{\langle ij \rangle, \sigma} c^\dagger_{i\sigma} c_{j\sigma} + \sum_i c^\dagger_i \left( \mathbf{S}_i \cdot \boldsymbol{\sigma} \right) c_i, \tag{2}$$

where the magnitude of $\mathbf{S}_i$ is fixed while its orientation is randomly distributed from site to site. Figure S4 compares the resulting unfolded spectral weight for a random spin configuration with the ferromagnetic case where all spins are aligned. Interestingly, the loss of long-range magnetic order does not lead to a simple closure of the exchange splitting, but instead produces substantial spectral broadening over the entire spectrum.

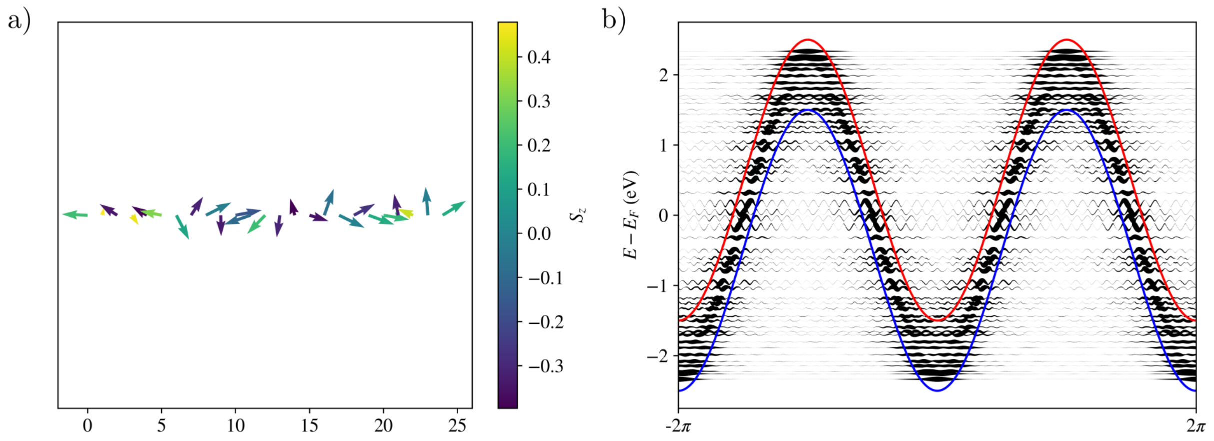


FIG. S4. a) Visualization of the chosen random arrangement of the spins with $N = 25$ sites, with arrows indicating $x$ and $y$ components of the spins and colors for the $S_{i,z}$. In (b) blue and red correspond to the band structure of a ferromagnetic 1D chain (all spins aligned), while black shows the unfolded spectral weight for a random spin configuration along the 25-site supercell. In the plots $|S_i| = 0.5$ and $t = -1$ eV

## V. COMPLEMENTARY DATA AND SIMULATIONS

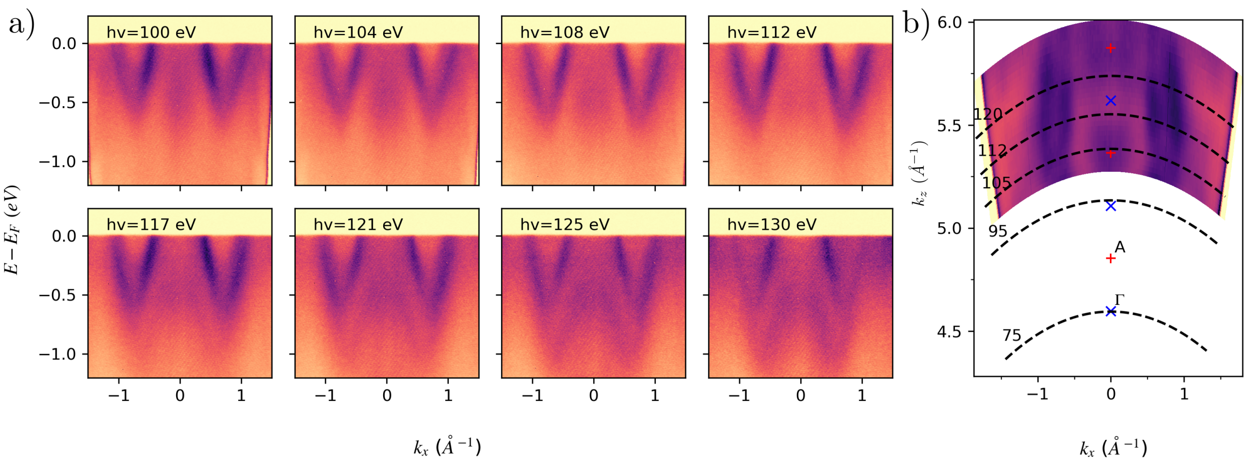


FIG. S5. a) In-plane dispersion along the ΓK direction at different photon energy. b) Fermi surface in the ΓKA plane reporting different lines indicating constant photon energy curves.

In Fig. S5 panel a we report the in-plane dispersion along the ΓK direction at different photon energy and in panel b the Fermi-surface in the ΓKA with photon energy ranging from 100 to 130 eV.

To understand the in-plane dispersion of the Fe-$d$-Ta-$d$ bands as a function of the out-of-plane momentum, we plot the band structure along the parallel direction as a function of the out-of-plane momentum (see Fig. S6). To clearly distinguish the Fe hybridized bands, the size of the points is proportional to the Fe-$d$ character of the band. As reported in Fig. 4, while the dispersion along $k_z$ is nearly linear around the Fermi level, along $k_{||}$ is parabolic as evident by the cuts at different $k_z$'s in Fig. S6. This strongly anisotropic behavior is typical of semi-Dirac materials where the dispersion along different $k$ directions is qualitative different [27–30]. In particular, in our 3D case, the energy dispersion near the Fermi level can be written as $E(k_x, k_y, k_z) = k_x^2/2m_1 + k_y^2/2m_2 + v_F k_z$ with $m_1$ and $m_2$ two different effective masses.

In Fig. S7, panel a, we present the simulated ARPES intensity at $k_z = \pi/c$ together with the corresponding unfolded band structure along the high-symmetry lines in the AHL plane. When comparing this unfolded dispersion with that in the ΓKM plane, we find that the splittings associated with the 2H stacking symmetry disappear: the bands that were previously split now become degenerate in the AHL plane. Importantly, the Zeeman splitting of the Ta $d$-derived bands induced by ferromagnetic Fe intercalation remains intact.

Finally, in Fig. S8, we show a comparison of the $k_z$ dispersion acquired with circular right (CR) and circular left light (CL) , showing no substantial differences. We superimposed to the SX-ARPES spectrum the out-of-plane dispersion of pristine 2H-$TaS_2$ to directly demonstrate how the out-of-plane dispersion is strongly modified by the Fe intercalant atom.

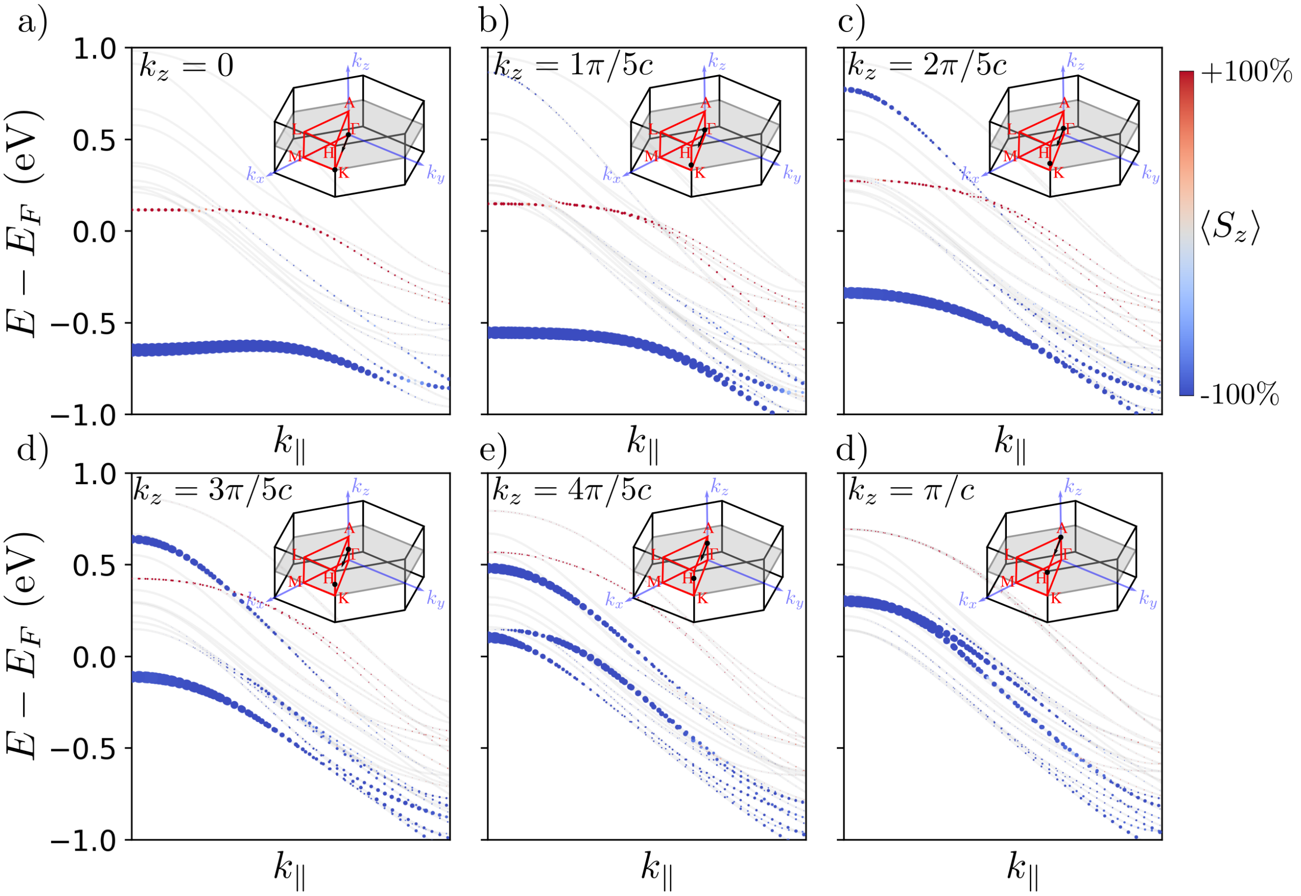


FIG. S6. In-plane dispersion at different $k_z$. We also report the Brillouin Zone and the path chosen for the band structure. The size of the points is proportional to the Fe-$d$ character, while the color is the spin-polarization.

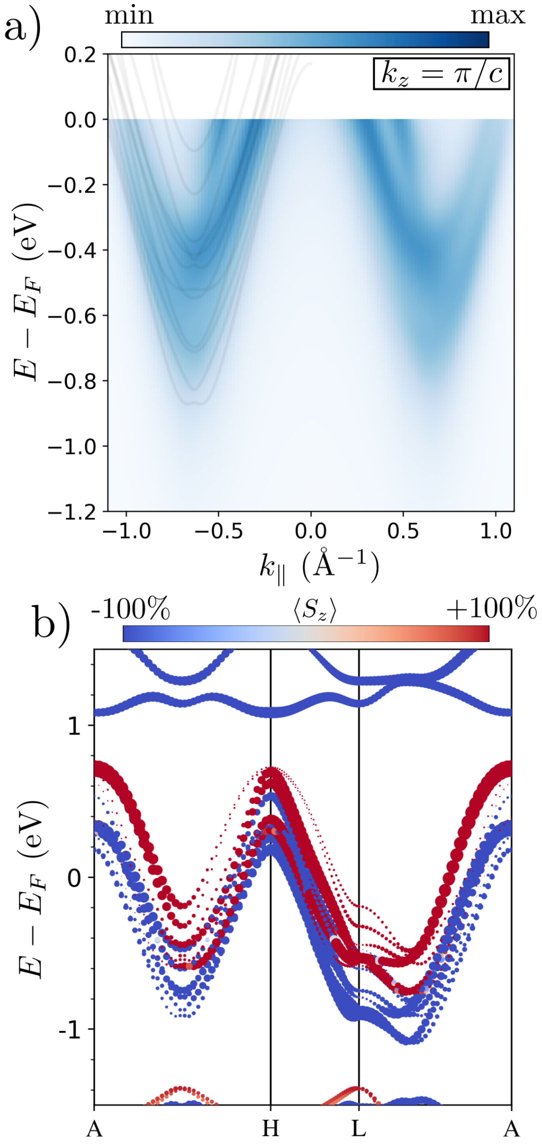


FIG. S7. a) Simulation of the ARPES intensity. On the left, we superimposed the band structure calculated using the PBE+$U$+$SOC$ functional. b) Unfolded and spin-polarized band structure. The size of the point are proportional to the weight of the unfolding, while the colormap is for the spin polarization $\langle S_z \rangle$ in the AHL plane.

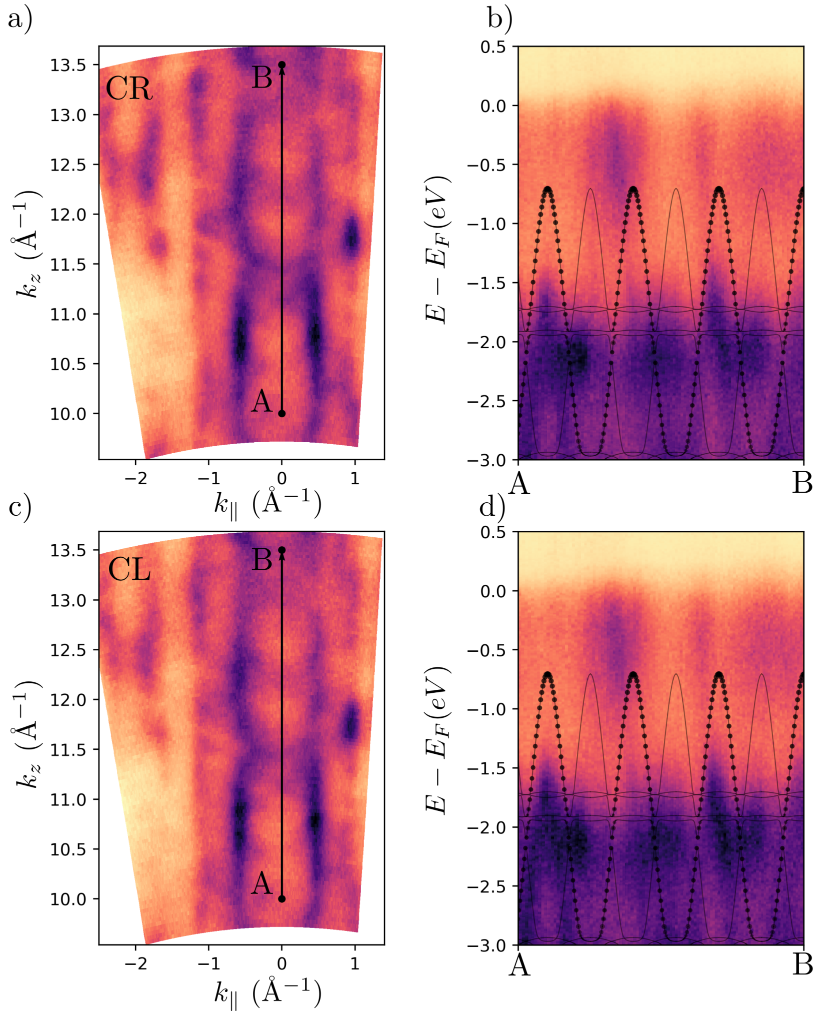


FIG. S8. a) Fermi surface in the ΓAK plane using CR and b) corresponding band structure at $(k_x, k_y)$=0 in which we superimpose band structure of the pristine 2H-$TaS_2$. The same for CL light in c) and d).

[1] G. Kresse and J. Hafner, "Ab initio molecular dynamics for liquid metals," Phys. Rev. B **47**, 558–561 (1993).

[2] G. Kresse and D. Joubert, "From ultrasoft pseudopotentials to the projector augmented-wave method," Phys. Rev. B **59**, 1758–1775 (1999).

[3] John P. Perdew, Kieron Burke, and Matthias Ernzerhof, "Generalized gradient approximation made simple," Phys. Rev. Lett. **77**, 3865–3868 (1996).

[4] Matteo Cococcioni and Stefano de Gironcoli, "Linear response approach to the calculation of the effective interaction parameters in the LDA + U method," Phys. Rev. B **71**, 035105 (2005).

[5] Yiwei Hu, Qiaoyan Hao, Baichuan Zhu, Biao Li, Zhan Gao, Yan Wang, and Kaibin Tang, "Toward exploring the structure of monolayer to few-layer $TaS_2$ by efficient ultrasound-free exfoliation," Nanoscale Research Letters **13** (2018), 10.1186/s11671-018-2439-z.

[6] J Dijkstra, P J Zijlema, C F van Bruggen, C Haas, and R A de Groot, "Band-structure calculations of $Fe_{1/3}TaS_2$ and $Mn_{1/3}TaS_2$, and transport and magnetic properties of $Fe_{0.28}TaS_2$," *Journal of Physics : Condensed Matter* **1**, 6363–6379 (1989).

[7] Sebastian Mangelsen, Julian Hansen, Peter Adler, Walter Schnelle, Wolfgang Bensch, Sergiy Mankovsky, Svitlana Polesya, and Hubert Ebert, "Large anomalous Hall effect and slow relaxation of the magnetization in $Fe_{1/3}TaS_2$," The Journal of Physical Chemistry C **124**, 24984–24994 (2020).

[8] Aliaksandr V. Krukau, Oleg A. Vydrov, Artur F. Izmaylov, and Gustavo E. Scuseria, "Influence of the exchange screening parameter on the performance of screened hybrid functionals," The Journal of Chemical Physics **125** (2006), 10.1063/1.2404663.

[9] J. Paier, M. Marsman, K. Hummer, G. Kresse, I. C. Gerber, and J. G. Ángyán, "Screened hybrid density functionals applied to solids," The Journal of Chemical Physics **124** (2006), 10.1063/1.2187006.

[10] Jochen Heyd, Gustavo E. Scuseria, and Matthias Ernzerhof, "Hybrid functionals based on a screened Coulomb potential," The Journal of Chemical Physics **118**, 8207–8215 (2003).

[11] Luigi Camerano, Dario Mastrippolito, Debora Pierucci, Ji Dai, Massimo Tallarida, Luca Ottaviano, Gianni Profeta, and Federico Bisti, "Darkness in interlayer and charge density wave states of 2H-$TaS_2$," Phys. Rev. B **111**, L121112 (2025).

[12] Volker Blum, Ralf Gehrke, Felix Hanke, Paula Havu, Ville Havu, Xinguo Ren, Karsten Reuter, and Matthias Scheffler, "Ab initio molecular simulations with numeric atom-centered orbitals," Computer Physics Communications **180**, 2175–2196 (2009).

[13] Xinguo Ren, Patrick Rinke, Volker Blum, Jürgen Wieferink, Alexandre Tkatchenko, Andrea Sanfilippo, Karsten Reuter, and Matthias Scheffler, "Resolution-of-identity approach to Hartree–Fock, hybrid density functionals, RPA, MP2 and GW with numeric atom-centered orbital basis functions," New Journal of Physics **14**, 053020 (2012).

[14] Jeremy P. Allen and Graeme W. Watson, "Occupation matrix control of d- and f-electron localisations using dft + u," Phys. Chem. Chem. Phys. **16**, 21016–21031 (2014).

[15] Andrea Damascelli, Zahid Hussain, and Zhi-Xun Shen, "Angle-resolved photoemission studies of the cuprate superconductors," Rev. Mod. Phys. **75**, 473–541 (2003).

[16] Jonathan A. Sobota, Yu He, and Zhi-Xun Shen, "Angle-resolved photoemission studies of quantum materials," Rev. Mod. Phys. **93**, 025006 (2021).

[17] Simon Moser, "An experimentalist's guide to the matrix element in angle resolved photoemission," Journal of Electron Spectroscopy and Related Phenomena **214**, 29–52 (2017).

[18] Yoonah Chung, Minsu Kim, Yeryn Kim, Seyeong Cha, Joon Woo Park, Jeehong Park, Yeonjin Yi, Dongjoon Song, Jung Hyun Ryu, Kimoon Lee, Timur K. Kim, Cephise Cacho, Jonathan Denlinger, Chris Jozwiak, Eli Rotenberg, Aaron Bostwick, and Keun Su Kim, "Dark states of electrons in a quantum system with two pairs of sublattices," Nature Physics (2024), 10.1038/s41567-024-02586-x.

[19] Eric L. Shirley, L. J. Terminello, A. Santoni, and F. J. Himpsel, "Brillouin-zone-selection effects in graphite photoelectron angular distributions," Phys. Rev. B **51**, 13614–13622 (1995).

[20] M. W. Haverkort, M. Zwierzycki, and O. K. Andersen, "Multiplet ligand-field theory using Wannier orbitals," Phys. Rev. B **85**, 165113 (2012).

[21] Y. Lu, M. Höppner, O. Gunnarsson, and M. W. Haverkort, "Efficient real-frequency solver for dynamical mean-field theory," Phys. Rev. B **90**, 085102 (2014).

[22] M. W. Haverkort, G. Sangiovanni, P. Hansmann, A. Toschi, Y. Lu, and S. Macke, "Bands, resonances, edge singularities and excitons in core level spectroscopy investigated within the dynamical mean-field theory," Europhysics Letters **108**, 57004 (2014).

[23] M Sacchi, N Jaouen, H Popescu, R Gaudemer, J M Tonnerre, S G Chiuzbaian, C F Hague, A Delmotte, J M Dubuisson, G Cauchon, B Lagarde, and F Polack, "The SEXTANTS beamline at SOLEIL: a new facility for elastic, inelastic and coherent scattering of soft x-rays," J. Phys. Conf. Ser. **425**, 072018 (2013).

[24] Sorin G Chiuzbăian, Coryn F Hague, Antoine Avila, Renaud Delaunay, Nicolas Jaouen, Maurizio Sacchi, François Polack, Muriel Thomasset, Bruno Lagarde, Alessandro Nicolaou, *et al.*, "Design and performance of AERHA, a high acceptance high resolution soft x-ray spectrometer," Rev, of Sci. Instrum. **85**, 043108 (2014).

[25] Alessandro Nicolaou, Victor Pinty, Fabrice Marteau, Jean-Michel Dubuisson, Patrick Rommeluere, Eric Dupuy, Francois Bouvet, Dominique Corruble, Conny Sathe, Marcus Agaker, Jean-Eric Rubensson, Mohamed Nouna, Pascal Goy, Stephan Lorcy, and Christian Herbeaux, "15th SOLEIL Users Meeting," `https://www.synchrotron-soleil.fr/en/file/12497/`

download?token=FX_uM-Vm (2020), accessed: 2024-12-20.

[26] Na Qin, Cheng Chen, Shiqiao Du, Xian Du, Xin Zhang, Zhongxu Yin, Jingsong Zhou, Runzhe Xu, Xu Gu, Qinqin Zhang, Wenxuan Zhao, Yidian Li, Sung-Kwan Mo, Zhongkai Liu, Shilei Zhang, Yanfeng Guo, Peizhe Tang, Yulin Chen, and Lexian Yang, "Persistent exchange splitting in the chiral helimagnet $cr_{1/3}nbs_2$," Phys. Rev. B **106**, 035129 (2022).

[27] Yinming Shao, Seongphill Moon, A.N. Rudenko, Jie Wang, Jonah Herzog-Arbeitman, Mykhaylo Ozerov, David Graf, Zhiyuan Sun, Raquel Queiroz, Seng Huat Lee, Yanglin Zhu, Zhiqiang Mao, M.I. Katsnelson, B. Andrei Bernevig, Dmitry Smirnov, Andrew J. Millis, and D.N. Basov, "Semi-Dirac fermions in a topological metal," Physical Review X **14** (2024), 10.1103/physrevx.14.041057.

[28] Petra Dietl, Frédéric Piéchon, and Gilles Montambaux, "New magnetic field dependence of Landau levels in a graphenelike structure," Physical Review Letters **100** (2008), 10.1103/physrevlett.100.236405.

[29] Victor Pardo and Warren E. Pickett, "Half-metallic semi-Dirac-point generated by quantum confinement in $TiO_2/VO_2$ nanostructures," Physical Review Letters **102** (2009), 10.1103/physrevlett.102.166803.

[30] S. Banerjee, R. R. P. Singh, V. Pardo, and W. E. Pickett, "Tight-binding modeling and low-energy behavior of the semi-Dirac point," Physical Review Letters **103** (2009), 10.1103/physrevlett.103.016402.
